# Synergistic effects of copper and ethylene on resveratrol synthesis in peanuts

**DOI:** 10.1002/fsn3.2117

**Published:** 2021-01-19

**Authors:** Xuefeng Bao, Guolin Lin, Xuan Dong, Xinyu Ma, Xiaori Han, Hui Zhang

**Affiliations:** ^1^ College of Land and Environment Shenyang Agricultural University Shenyang China

**Keywords:** copper ion and ethylene, interaction effect, secondary metabolites, synthesis of stilbene

## Abstract

This study aimed to investigate the effects of exogenous copper and ethylene on resveratrol biosynthesis in peanut buds. In this study, different concentrations of copper sulfate and ethephon were used to induce peanut bud roots. Resveratrol content was determined using high‐performance liquid chromatography (HPLC). The interaction effects of the two inducers were analyzed using regression and response surface methodology. Results showed that resveratrol biosynthesis increased with the increase in copper sulfate concentration. Resveratrol synthesis showed an increasing trend when the concentration of ethephon was from 5 to 7 mmol/L. The resveratrol content reached a maximum value of 7.7 μg/g when the concentration of ethephon was 7 mmol/L and then decreased with an increase in ethephon. Response surface analysis revealed that the combination of 0.1 mmol/L copper sulfate and 5 mmol/L ethephon was effective and resulted in the induction of resveratrol biosynthesis in peanut buds.

## Graphical abstract

For this research, the change of copper sulfate concentration is from 0.1 to 0.5 mmol/L, and the change of ethephon concentration is from 2 to 9 mmol/L. In such an induction range, when inducing resveratrol biosynthesis in peanut bud, in more cases, the two elicitors showed synergistic effect and increased resveratrol biosynthesis.

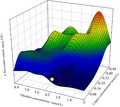

## 1 INTRODUCTION

Resveratrol is a polyphenol plant secondary metabolite that has been found in more than 100 plants, such as peanuts, grapes, and *Polygonum cuspidatum* (Li Xiankuan et al., 2016; Takaota, 1940). It has attracted much attention from researchers because it has outstanding biological activities, including anticancer (Jang et al., 1997), antiplatelet agglutination (Orsini et al., 1997), antioxidant (Gülçin, 2010), and antiviral activities (Delmas et al., 2011; Shazib et al., 2009; Wang et al., 2010).

The synthesis of secondary metabolites in plants is regulated by elicitors and signaling materials. Both biological (Ahn et al., 2015; Assia et al., 2006; Deng et al., 2016) and abiotic (Adrian et al., 2000) stimulation can lead to resveratrol accumulation. Copper (Aziz et al., 2006) ions can stimulate the accumulation of resveratrol in peanuts, grapes, and other plants. Chung et al. (2003) reported a resveratrol content that was 2–9 times greater than that of the control group after 24‐hr treatment with salicylic acid, jasmonate, and ethylene.

Copper ions are a cofactor for the ethylene receptor. Ethylene signals cannot be effectively induced without copper ions (Burg & Burg, 1967). However, it has not been reported whether the synergistic stimulation by copper and ethylene can promote the accumulation of resveratrol in peanut buds. Thus, it is necessary to study the effects of copper and ethylene on resveratrol biosynthesis in peanut buds and determine the effective concentrations of the two elicitors.

## 2 MATERIALS AND METHODS

### 2.1 Germination of peanut seeds

Haihua No. 1 is a cultivated peanut (*Arachis hypogaea* Linn.) variety that was produced via the hybridization of two cultivated peanuts in 1977 by the Agricultural Science Team of Heigu Village, Haiyang City, Shandong Province, China. Its male parent is Linhua No. 1, and its female parent is Baisha No. 171. Haihua No. 1 is an excellent peanut cultivar that is widely planted in Shandong Province, China, and the roots of peanut seedlings contain high levels of resveratrol.

In this experiment, we used Haihua No. 1; 4,500 peanut seeds were washed three times with distilled water and then soaked in distilled water at 35°C for 8 hr (Yu et al., 2016). During the soaking period, water absorption by the seeds was recorded in each hour and distilled water was added to ensure sufficient water. After 8 hr of soaking in the water bath, seeds with dropped seed coats, ruptured hypocotyls and cotyledons, or those that were unable to fully absorb water were removed. Peanut seeds were evenly spread on a 35 cm × 27 cm culture tray that was sterilized with 95% alcohol and covered with a sterilized towel, which had been soaked in distilled water to ensure that the seeds were moistened. The seeds were incubated at 29°C without light for 3 days. Peanut buds that were approximately 2–3 cm long were randomly selected as one sample. They were placed in a small plastic cup that contained 70 ml of water and covered with a planting basket. The selected peanut buds were incubated at 25°C with illumination for 10 hr, and at 15°C without illumination for 14 hr over a total of 3 days. The slurry in the plastic cup with peanut buds was replaced with distilled water daily, and the peanut buds infected by bacteria were removed. We selected the peanut buds with fibrous roots, and roots that were 4–5 cm in length and 4–5 g in weight as the experimental materials.

### 2.2 Treatment

The molecular weight of copper sulfate pentahydrate (purchased from Sinopharm Chemical Reagent Co., Ltd.) was 250 g/mol, and the molecular weight of ethephon (purchased from Shanghai YuanYe Biotechnology Co., Ltd.) was 144.5 g/mol. The two types of drugs were accurately weighed with an electronic balance, and distilled water was added to volumetric flasks for solution preparation. The volume of the treatment solution was 70 ml to allow every peanut bud root to be fully submerged in the treatment solution. Therefore, for every copper–ethylene interactive treatment, 35 ml of the treatment solution at double concentration was measured out using a measuring cylinder. The solution was poured into a plastic cup and mixed evenly with a glass rod.

There were two types of treatments.

Type A consisted of single‐solution treatments:

- For the ethylene treatments without copper, the ethephon concentrations were 0, 2, 4, 5, 6, 7, 8, and 9 mmol/L.
- When Cu was used with ethylene, the copper sulfate concentrations were 0, 0.1, 0.2, 0.3, 0.4, and 0.5 mmol/L.

Type B consisted of interactive treatments. For each interactive treatment, a total of 35 ml of copper sulfate and ethephon solution at twice the concentration of the corresponding single treatment was measured out into a plastic cup using a measuring cylinder and mixed evenly with a glass rod. All concentrations and labels for the interactive treatments are shown in Table 1.

**TABLE 1:** Solution concentrations for the interactive treatments

Laboratory processing label	Copper ion concentration (mmol/L)					
	0	0.1	0.2	0.3	0.4	0.5
Ethylene concentration (mmol/L)						
0	CK	Cu1	Cu2	Cu3	Cu4	Cu5
2	E1	E1‐Cu1	E1‐Cu2	E1‐Cu3	E1‐Cu4	E1‐Cu5
4	E2	E2‐Cu1	E2‐Cu2	E2‐Cu3	E2‐Cu4	E2‐Cu5
5	E3	E3‐Cu1	E3‐Cu2	E3‐Cu3	E3‐Cu4	E3‐Cu5
6	E4	E4‐Cu1	E4‐Cu2	E4‐Cu3	E4‐Cu4	E4‐Cu5
7	E5	E5‐Cu1	E5‐Cu2	E5‐Cu3	E5‐Cu4	E5‐Cu5
8	E6	E6‐Cu1	E6‐Cu2	E6‐Cu3	E6‐Cu4	E6‐Cu5
9	E7	E7‐Cu1	E7‐Cu2	E7‐Cu3	E7‐Cu4	E7‐Cu5

Note

The table shows the concentrations of the peanut bud root treatment solution and the labels corresponding to the treatments. There were 48 treatments, with (a) Eight ethylene concentration gradients (0, 2, 4, 5, 6, 7, 8, and 9 mmol/L) for treatments without copper sulfate. (b) Six copper concentration gradients (0, 0.1, 0.2, 0.3, 0.4, and 0.5 mmol/L) for treatments with ethylene. (c) Thirty‐five interactive treatments. The data in this table consist of labels for the treatments corresponding to the concentration of the ethylene and copper interaction, where **E** stands for ethylene and **Cu** for copper.

Each treatment contained four biological repetitions, the volume of each cup of treatment solution was 70 ml, and each cup contained five peanut seeding buds. The treatment duration was 48 hr.

### 2.3 The *t*‐resveratrol extraction method for treated peanut root buds

The peanut roots were removed after 48 hr of treatment. They were then weighed using a balance and placed in a grinding bowl, and a cup of liquid nitrogen was used to rapidly freeze them. The frozen root samples were quickly placed in a ball mill and ground for 15 s at 6.47 *g*. After grinding, the root sample was scraped out using an iron spoon. The ground slurry was placed in a centrifuge tube, and 10 ml of extraction solution (80% methanol) was added. After placing the cover, the tube was gently shaken, placed into the centrifuge tube frame, and shaken using a constant‐temperature shaker for 60 min at 280 rpm and 40°C. The concussion liquid was ultrasonicated for 180 min at 50°C and then centrifuged for 15 min at 3500 *g*. The centrifuged sample was slowly filtered into a 50‐ml beaker, and the filtrate was further filtered into a 2‐ml brown vial using an organic syringe filter (pore diameter: 0.22 μm). The filtrate was stored at −40°C before high‐performance liquid chromatography (HPLC) analysis.

### 2.4 HPLC analysis

An HPLC instrument (Agilent, 1100 series), which was equipped with a QuatPump quaternary pump (DE11115754), a DAD (DE11113430) detector (photoelectric diode array detection, detection wavelength of 306 nm), and a chromatographic column (Spursil C18, 5 μm, 250 × 4.6 mm) with a porosity of 5 μm (for water), was used to determine the *t*‐resveratrol.

The elution method was isocratic elution, with a passing rate of 100% in 15 min. The eluent components included deionized water, methanol (≥99.9%, Sigma‐Aldrich), and acetonitrile (assay [bygc] ≥99.9%, ANPEL Scientific Instrument Co., Ltd.). The volume ratio of the eluent was deionized water (99.9%), methanol (99.9%), and acetonitrile = 50%, 20%, and 30%. The volume of the injected sample at each time point was 20 μl, the column temperature was 30°C, and the flow rate was set to 0.8 ml/min.

The qualitative and quantitative analyses of *t*‐resveratrol were based on the retention time and absorbance of the standard substance (the retention time was between 6.28 and 6.35 min, and the absorbance was measured at a wavelength of 306 nm). The standard curve for *t*‐resveratrol was based on the standard product of *t*‐resveratrol (purchased from Shanghai YuanYe Biotechnology Co., Ltd.), and the mother liquor was diluted in multiple proportions. A 5 mg sample was accurately weighed using an electronic balance, methanol was used as the solvent, and the volume was fixed in a 50‐ml volumetric flask to prepare 100 μg/ml of the mother liquor. According to the requirements of this test, the dilution was 0.1, 0.5, 1, 5, 10, or 50 μg/ml. After these standard samples were tested by HPLC, the standard curve was drawn using the integral values of the peak area corresponding to *t*‐resveratrol and standard *t*‐resveratrol sample concentrations.

### 2.5 Liquid chromatogram of the resveratrol standard and the treated samples

See Figure 1.

**FIGURE 1:**
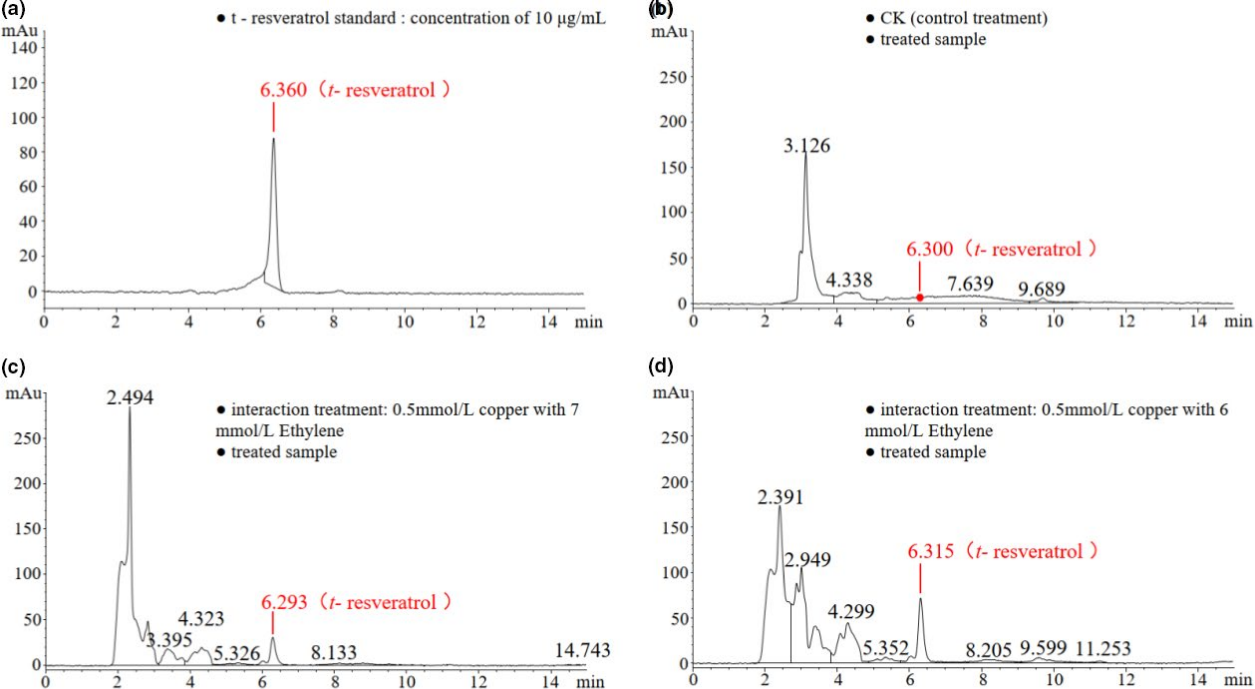
Liquid chromatogram of the resveratrol standard and treated samples

### 2.6 Statistical methods

The concentration of *t*‐resveratrol in each 48‐hr treated sample was converted using a standard curve, and the *t*‐resveratrol content in the peanut seeding buds was converted using the root sample weight and a volume of 80% methanol at the time of sampling. The converted unit (μg/g FW) was then statistically analyzed.

Treated fresh peanut root samples were tested using HPLC, and the content of resveratrol in each sample was calculated using a standard curve. All data were processed using Microsoft Excel 2016. A one‐way analysis of variance (followed by Duncan's multiple comparison method) was performed using SAS 9.2 statistical analysis software. The analysis results were plotted as histograms and scatter plots using Origin 2019. The data used to draw the response surface map using SigmaPlot 12.2 were the average value of each treatment.

## 3 RESULTS

### 3.1 Resveratrol change trend under the single‐factor treatments

#### 3.1.1 Resveratrol concentration trends under the different copper ion treatments

**FIGURE 2:**
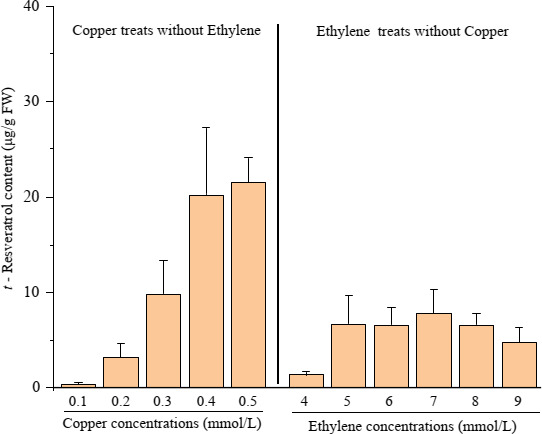
Peanut bud resveratrol content at the different copper and ethephon levels. (a) Haihua No. 1 (a peanut cultivar) seeds after soaking for 8 hr (in a constant‐temperature water bath at 35°C) were cultured in a constant‐temperature incubator for 6 days (during the first 3 days, they were covered with towels in trays at 29°C. During the latter 3 days, the peanut bud roots were soaked in a plastic cup containing distilled water at 25°C for 10 hr and 15°C for 14 hr each day). (b) After the 6‐day seed treatment, the peanut buds were treated with copper sulfate solution (copper concentration gradient: 0.1–0.5 mmol/L) and ethephon solution (ethylene concentration gradient: 4–9 mmol/L) for 48 hr, and each treatment included four biological repetitions. (c) After treatment, the content of t‐resveratrol in the roots of peanut buds was determined by HPLC using fresh root samples. (d) The data are expressed as the mean ± standard error. The *t*‐resveratrol under the 2 mmol/L ethylene treatment was 0; therefore, its value is not shown in this figure

The peanut buds were treated with copper ions at different concentrations for 48 hr, and resveratrol synthesis in the roots of the peanut buds was compared. Resveratrol synthesis was 0.33 μg/g when treated with 0.1 mmol/L copper ions and 3.13 μg/g in the root of the peanut bud when treated with 0.2 mmol/L copper ions. It increased by approximately eight times compared with that in the 0.1 mmol/L copper ion treatment. The resveratrol synthesis amounts were 9.78, 20.23, and 21.44 μg/g at 0.3, 0.4, and 0.5 mmol/L, respectively. Resveratrol synthesis increased as the treatment concentrations increased (Figure 2).

#### 3.1.2 Resveratrol content trends at the different ethephon concentrations

The peanut buds were treated with ethephon at different concentrations for 48 hr, and resveratrol synthesis in the roots of peanut buds was compared. Resveratrol was not detected in the roots of peanut buds treated with 0 and 2 mmol/L ethephon but was 1.34 μg/g when treated with 4 mmol/L ethephon. The resveratrol content increased to 6.65 μg/g when treated with 5 mmol/L ethephon, which was four times higher than that in the 4 mmol/L ethephon treatment. There was no significant difference in resveratrol synthesis when the concentration increased. The resveratrol synthesis in the roots of peanut buds treated with 6, 7, 8, and 9 mmol/L was 6.51, 7.77, 6.58, and 4.7 μg/g, respectively. Resveratrol synthesis increased at first and then decreased as the treatment concentrations increased (Figure 2).

### 3.2 Ethephon and Cu^2+^ interactions with resveratrol biosynthesis in peanut buds

#### 3.2.1 Effect of Cu^2+^ on resveratrol biosynthesis in peanut buds at the different ethephon concentrations

There was a significant linear relationship between resveratrol content and the induction of copper ions when the ethephon concentration increased from 0 to 5 mmol/L. Under every ethephon condition, resveratrol synthesis increased as the copper ion concentration increased. There was a significant linear relationship between resveratrol content and the induction of copper ions when the concentration of ethephon increased to 6 and 7 mmol/L. Under every ethephon condition, resveratrol synthesis first increased and then decreased as the copper ion concentration increased.

**FIGURE 3:**
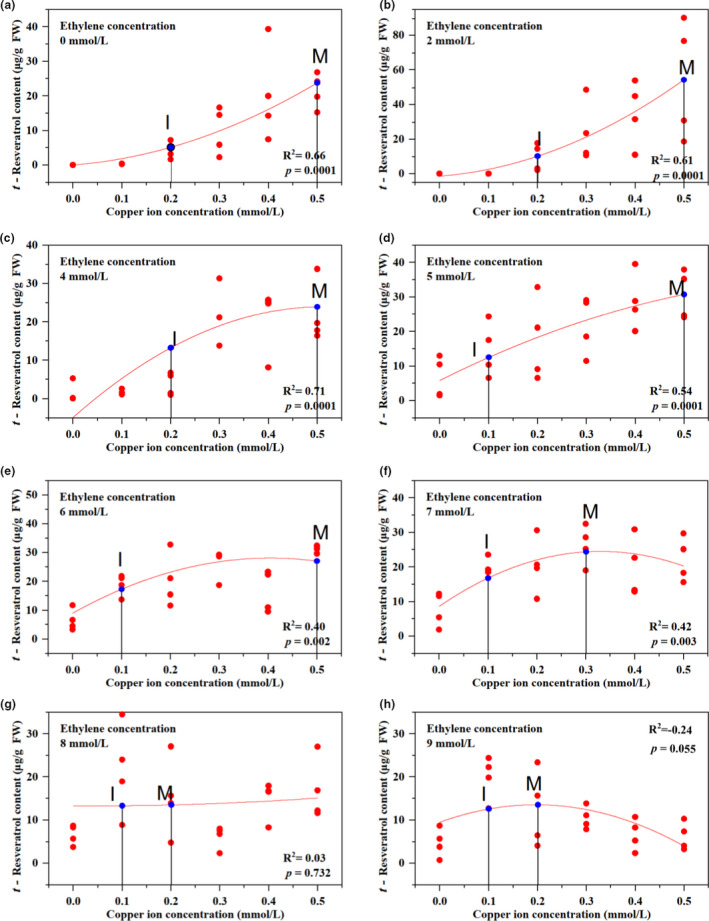
Resveratrol biosynthesis in peanut buds at different ethylene concentrations. (a) Haihua No. 1 (a peanut cultivar) seeds after soaking for 8 hr (in a constant‐temperature water bath at 35°C) were cultured in a constant‐temperature incubator for 6 days (during the first 3 days, they were covered with towels in trays at 29°C. During the latter 3 days, the peanut bud roots were soaked in a plastic cup containing distilled water at 25°C for 10 hr and 15°C for 14 hr each day). (b) After the 6‐day seed treatment, the peanut buds were treated with copper sulfate solution (copper concentration gradient: 0.1–0.5 mmol/L) and ethephon solution (ethylene concentration gradient: 4–9 mmol/L) for 48 hr, and there were four biological repetitions for each treatment. (c) After treatment, the *t*‐resveratrol content in the peanut bud roots was determined by HPLC analysis of fresh root samples. (d) In each scatter chart: (i) Data points represent the resveratrol biosynthesis by each biological repeat under the corresponding treatments. (ii) Point **I** represents the effective initial concentration, and point **M,** the maximum concentration

There was no significant linear relationship between resveratrol content and the induction of copper ions when the ethephon concentration increased to 8 mmol/L. The synthesis of resveratrol from peanut buds decreased as the copper ion levels increased when the ethephon concentration was greater than 8 mmol/L (e, f, g, and h in Figure 3).

**FIGURE 4:**
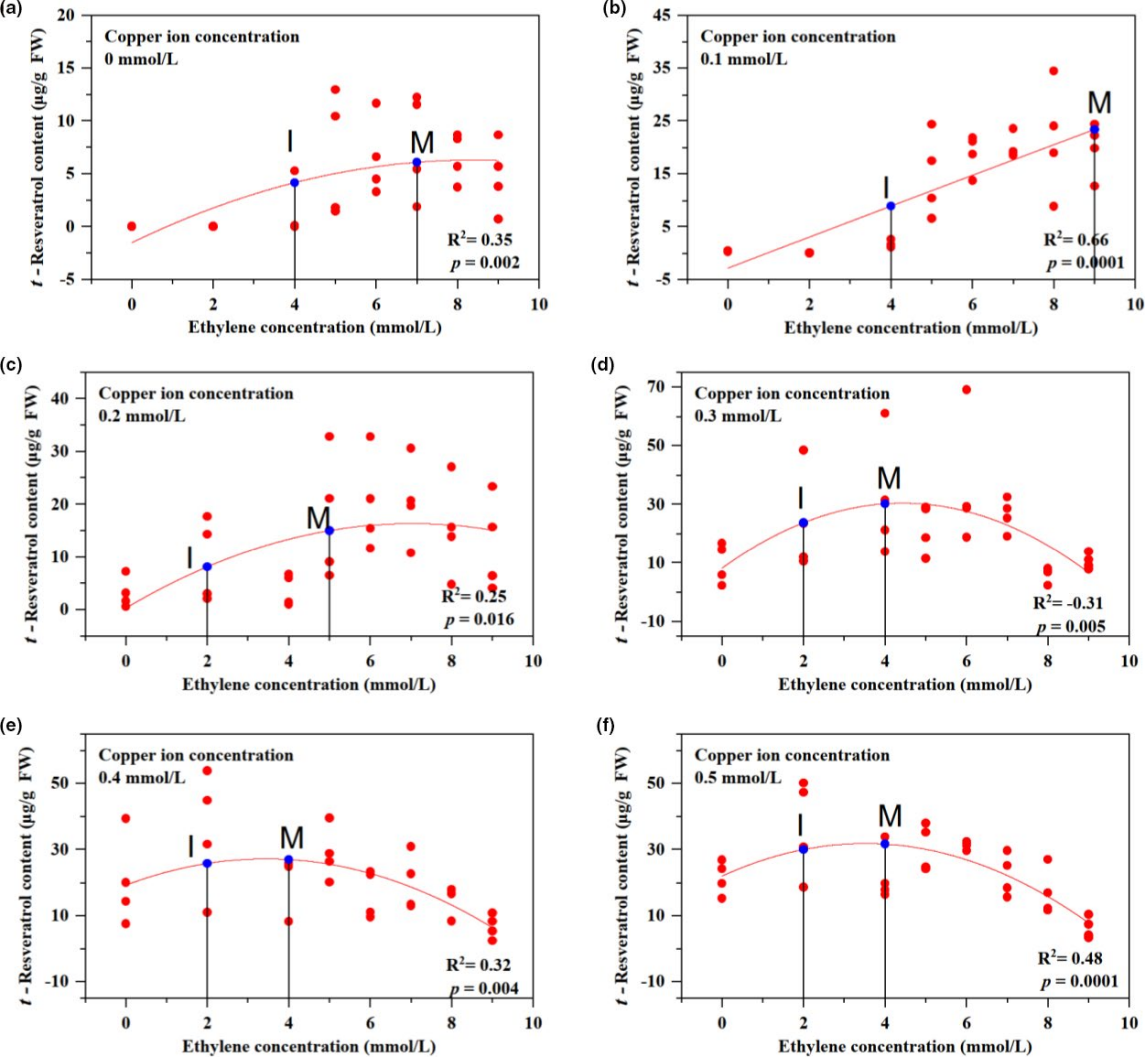
Resveratrol biosynthesis in peanut buds at different copper concentrations. (a) Haihua No. 1 (a peanut cultivar) seeds, after soaking for 8 hr (in a constant‐temperature water bath at 35°C), were cultured in a constant‐temperature incubator for 6 days (during the first 3 days, they were covered with towels in trays at 29°C. During the latter 3 days, the peanut bud roots were soaked in a plastic cup containing distilled water at 25°C for 10 hr and 15°C for 14 hr each day). (b) After the 6‐day seed treatment, the peanut buds were treated with ethephon solution (ethylene concentration gradient 4–9 mmol/L) for 48 hr. Individuals in each ethylene concentration group shared the same copper concentration to explore the resveratrol biosynthesis trend under the copper–ethylene interactive treatments. Each treatment had four biological repetitions. (c) After treatment, the *t*‐resveratrol content in the roots of peanut buds was determined by HPLC analysis of fresh root samples. (d) In each scatter chart: (i) Data points represent the resveratrol biosynthesis by each biological repeat under the corresponding treatment. (ii) Point **I** represents the effective initial concentration, and point **M** is the maximum concentration

The effective initial concentration of copper ions was 0.2 mmol/L when the ethephon concentration was 0, 2, and 4 mmol/L (a, b, and c in Figure 4). The effective initial concentration of copper ions decreased to 0.1 mmol/L when the ethephon concentration increased to 5, 6, and 7 mmol/L (d, e, and f in Figure 3).

### 3.3 Effect of ethephon on resveratrol biosynthesis in peanut buds under the different Cu^2+^ concentrations

**FIGURE 5:**
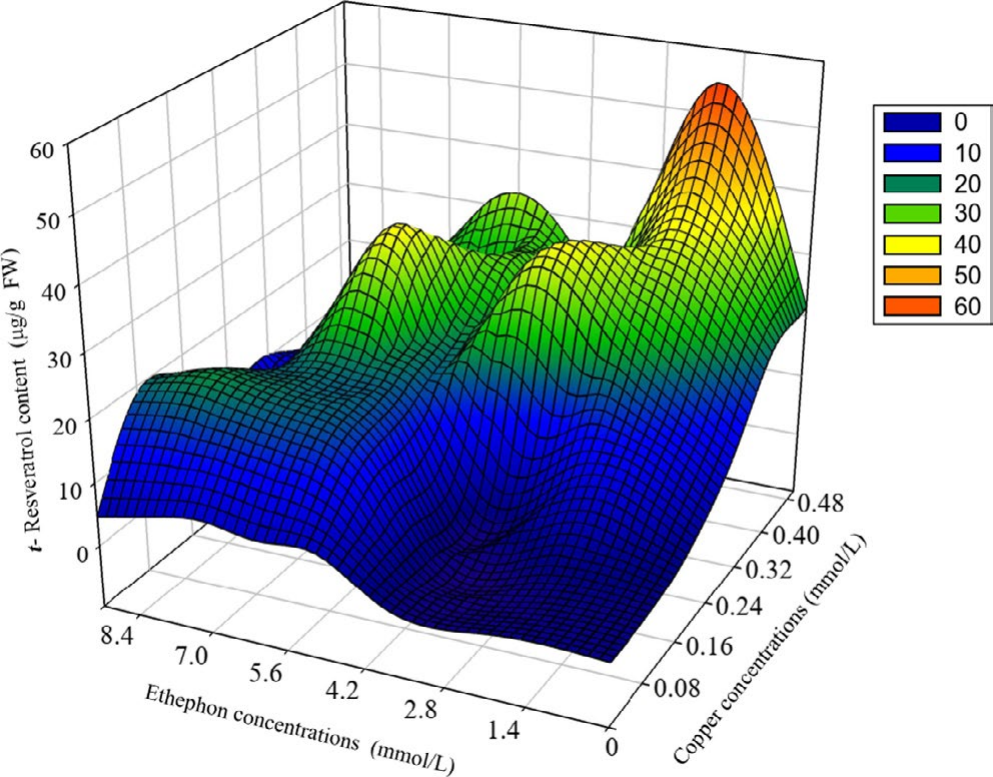
Resveratrol content under different copper and ethylene interactions. Figure 5 shows that the resveratrol content in peanut buds responds to different concentrations of copper–ethylene in the interaction treatments (48 hr). The mapping data for resveratrol biosynthesis are the average values of resveratrol biosynthesis for the four repetitions in each treatment. Bivariate polynomial regression: *Y* = 16.939 + 16.59*X*_1_ + 3.567*X*_2_ + 2.095*X*_1_ × *X*_1_ − 1.95*X*_1_ × *X*_2_ − 4.206*X*_2_ × *X*_2_, *X*_1_ represents the copper ion concentration, *X*_2_ represents the ethephon concentration, and *Y* represents the resveratrol concentration; *R* ^2^ = .7269

There was a significant linear relationship between resveratrol content and induction with ethephon at different concentrations under the single‐factor ethephon treatments. Resveratrol synthesis increased at first and then decreased (a in Figure 5). There was a good linear relationship between resveratrol content and ethephon levels when the copper ion concentration was 0.1 mmol/L. Resveratrol synthesis increased as the ethephon concentration increased (b in Figure 5). There was a linear relationship between resveratrol synthesis and ethephon concentration when the concentration of copper ions was 0.2–0.5 mmol/L. Resveratrol synthesis increased at first and then decreased (c, d, e, and f in Figure 4). The effective initial concentration of ethephon was 5 mmol/L when the copper ion concentration was 0 and 0.1 mmol/L (a and b in Figure 4).

### 3.4 Response surface analysis of the effects of different copper sulfate and ethephon concentrations on resveratrol synthesis in germinated peanuts

Resveratrol synthesis showed an increasing trend with the interaction between ethephon and copper ions when the ethephon content was less than 5 mmol/L and the copper ion concentration was 0.1–0.5 mmol/L. Resveratrol synthesis tended to be constant for the interaction between ethephon and copper ions when the ethephon concentration was 0.3–0.5 mmol/L and the copper ion concentration was 0.1–0.2 mmol/L. Resveratrol concentration increased or decreased with the interaction between ethephon and copper ions. When the concentration of ethephon is 5‐9 mmol/L and the concentration of copper ion is 0.2‐0.5 mmol/L, the biosynthesis of resveratrol changes greatly, but it does not show a simple upward or downward trend (Figure 5).

## 4 DISCUSSION

Previously, most studies have focused on peanut anti‐inflammatory, antioxidant, and other biological activities. However, there has been little research on the increase in resveratrol stimulated by metal ions. There have been a few studies on promoting resveratrol levels in peanuts by the synergetic induction of metal ions and hormones. This study could be used as a theoretical basis for “exploring the molecular mechanism of resveratrol synthesis from peanuts induced by copper ions and ethylene.”

In this experiment, peanut seedlings began to synthesize resveratrol at 0.1 mmol/L for a single factor. Peanut seedlings produced more resveratrol under stimulation conditions of 0.2 mmol/L copper ions (Figure 2). Resveratrol synthesis increased as the copper ion concentration increased.

In an experiment investigating resveratrol biosynthesis induced by a chitosan oligomer and copper sulfate solution, the optimum copper ion concentration was also 0.2 mmol/L, whereas resveratrol synthesis decreased when the copper ion concentration increased to 0.4 mmol/L (Aziz et al., 2006). In this experiment, resveratrol was induced by copper ions in peanut seedlings. The minimum effective copper ion concentration was 0.1 mmol/L.

Peanut seedlings began to synthesize resveratrol at the 4 mmol/L ethephon concentration. There was no significant difference in the resveratrol content of peanut seedlings when the ethephon concentration was 5–8 mmol/L. Resveratrol synthesis began to decrease when the ethephon concentration was 9 mmol/L. The results showed that the tolerance of peanut buds to ethephon ranged from 5 to 8 mmol/L. Resveratrol was induced by ethephon in peanut seedlings, and the effective ethephon concentration was 5 mmol/L (Figure 5).

An experiment investigating mechanical injury, herbicides, ultraviolet rays, and various hormones in different tissues of mature peanuts showed that the expression of the stilbene synthase gene mRNA was high when 5 mmol/L of ethephon was applied to peanut leaves for 24 hr, and the corresponding resveratrol biosynthesis was 2.18 μg/g FW (Chung et al., 2003). Additionally, it was notable that the biosynthesis of stilbene in peanut roots (approximately 2.1 μg/g) was higher than that of resveratrol in the leaves, pods, and seed coats. The results from this study showed that it is reasonable to select the roots of peanut seedlings for resveratrol determination.

In an experiment using fungi to infect peanuts, resveratrol was detected in an area close to the fungal infection after *Aspergillus flavus* infection for 24 hr (Sobolev, 2008). However, only after *A. flavus* infection for 48 hr could resveratrol biosynthesis be measured in areas distant from the fungal infection. The damage caused by copper ions to peanuts was less that that by a fungal infection; therefore, resveratrol biosynthesis in peanut seedlings treated with copper ions for 48 hr was detected by HPLC. In our previous experiments, resveratrol was not tested under 24‐hr copper treatment. Therefore, resveratrol biosynthesis in peanut seedling buds and roots caused by a response to elicitors may require at least 24 hr.

This induction concentration is consistent with an effective initial concentration of 5.54 mmol/L for resveratrol synthesis induced by ethephon at the young grape fruit stage (Dai Hongjun et al., 2014). This suggested that the resveratrol synthesis conditions may be the same for the peanut bud stage and grape fruit stage.

Copper ions and ethephon showed positive effects when the ethephon concentration was 2–5 mmol/L and the copper ion concentration was 0.1–0.5 mmol/L. The degree of fit reached more than 50% (b, c, and d in Figure 3). Copper ions and ethephon showed negative effects when the ethephon concentration was 6–9 mmol/L and the copper ion concentration was 0.1–0.5 mmol/L (e, f, g, and h in Figure 3). The results showed that the critical value for the ethephon interaction concentration was 5 mmol/L.

A recent study on ultraviolet rays and coumaric acid induced in grape leaves (Kiselev et al., 2019) showed that *p*‐coumaric acid induction (24 hr) could increase the expression of members of different stilbene synthase gene families by approximately 0.5–15 times. In UV ray–coumaric acid interactive induction (24 hr), the expression levels increased to a greater extent, and those of three gene family individuals increased by approximately 46–49 times. Coumaric acid can be used as a substrate for resveratrol biosynthesis, whereas ultraviolet rays could be regarded as an inducer with strong induction ability, which leads to stilbene synthase gene expression. Kiselev et al. (2019) showed that the expression level of different members of the stilbene synthase gene family was enhanced to varying degrees. However, some studies show no linear correlation between stilbene synthase gene expression levels and resveratrol biosynthesis (Chung et al., 2003). Thus, the increase in stilbene synthase gene expression level does not necessarily indicate an increase in resveratrol biosynthesis. Therefore, studying the interaction of different elicitors on resveratrol biosynthesis is an essential research topic that can reveal the secondary metabolic pathway at the metabolic level and will also create a physiological foundation for additional studies on the expression and transcriptional regulation of many synthetase genes in this pathway.

Copper ions and ethephon showed positive effects when the copper ion concentration was 0.1 mmol/L and the ethephon concentration was 4–9 mmol/L. The degree of fit could reach more than 66% (b in Figure 4). Copper ions and ethephon had negative effects when the copper ion concentration was 0.2–0.5 mmol/L and the ethephon concentration was 4–9 mmol/L (c, d, e, and f in Figure 5). The results showed that the critical value of the copper ion interaction concentration was 0.1 mmol/L.

## 5 CONCLUSION

The results from this study showed that the changes occurred when the copper sulfate concentration was 0.1–0.5 mmol/L and the ethephon concentration was 2–9 mmol/L. In such an induction range, when inducing resveratrol biosynthesis in peanut buds, the two elicitors generally showed synergistic effects and increased resveratrol biosynthesis. Among these concentrations, the best interaction combination was for 0.1 mmol/L copper ion concentration and 5 mmol/L ethephon concentration.

## Data Availability

The data were collected from the analysis and test center of Shenyang Agricultural University.
